# Health system innovations: adapting to rapid change

**DOI:** 10.1186/s12992-018-0347-8

**Published:** 2018-03-09

**Authors:** Gerald Bloom, Annie Wilkinson, Abbas Bhuiya

**Affiliations:** 1 Institute of Development Studies, University of Sussex, Brighton, BN1 9RE UK; 2 Icddr,b, Dhaka, Bangladesh

**Keywords:** Technological innovation, Organizational innovation, Change management, Universal health coverage, Low and middle-income countries

## Abstract

This paper introduces the Thematic Issue on Innovation in Health Systems in Low- and Middle-Income Countries.

A fundamental challenge for health systems is the need to adapt to changes in the patterns of health service need, scientific and technological developments, and the economic and institutional contexts within which providers of health services are embedded. This is especially true of many low and middle-income countries, where the pace of multiple and interconnected changes is breath-taking [1]. At a major meeting of UHC2030 in Tokyo in December 2017 several leaders in global health emphasized the potential role of technological innovations in making progress towards universal health coverage.

There is a growing body of literature on health systems innovation. A Web of Science search for “innovation” and “health systems” returned 54 results in 1999–2000, 86 in 2004–5, 159 in 2009–10 and 187 in 2014–15. In recent years there has been a very rapid increase in investment in innovations by bilateral donor agencies and health foundations. A major emphasis of this investment has been on relatively small-scale pilot projects aimed at a proof of an innovative technological or organizational concept. The outcomes, in terms of health system change or improved health outcomes have tended to be disappointing. This has led to calls for a systems (or systematic) approach towards stimulating the emergence and diffusion of innovations at scale.

Despite the growing interest in the potential contributions that a number of innovations can make towards improving access to health services, the dominant conceptualizations of health system development have tended to focus on the different “pillars” of a health system, while paying relatively little attention to the consequences for these systems of rapid change. However, new thinking is emerging. A number of publications now describe the health sector as a complex adaptive system and the focus of so-called “implementation research” is on the process of change, rather than the specific design of a particular intervention [2]. Others are exploring long-term historical processes of health system development. A notable example is a recent special issue of The Lancet [3] with a number of papers analyzing the management of health system innovation in Bangladesh over more than three decades.

The purpose of this thematic issue is to stimulate analyses of innovation in the context of complex and dynamic health systems. Although the focus is on low and middle-income countries, the papers have a wider geographic relevance. A number of rapidly growing low and middle-income countries are becoming global centers of technological and organizational innovation. The governments of these countries are under growing pressure to meet the health and health care needs of very large numbers of people whose incomes are rising above the poverty line and the innovations that emerge will soon have global impact. The expertise that governments of these countries are developing in the management of rapid change has important lessons for all governments.

The initial papers in this thematic issue present the findings of research by members of the Future Health Systems Consortium. It soon became clear that other researchers have been thinking along complementary lines. Members of the thematic working group on the private sector of Health Systems Global, an international network of health system researchers, offered to join the editorial team and we have decided to open the thematic issue to other papers relevant to the theme. We invite authors of papers to submit them for consideration by the thematic issue. And, we encourage people interested in this topic to participate in the work of the thematic working group by contacting us at psinhealth@gmail.com

## References

[1] Bloom G, Wolcott S (2013). Building institutions for health and health Systems in Contexts of rapid change. Soc Sci Med.

[2] Peters D, Adam T, Alonge O, Agyepong I, Tran N (2013). Implementation research: what it is and how to do it. Br Med J.

[3] The Lancet (2013) Bangladesh: Innovation for Universal Health Coverage November 2013.

